# Ethnic Variation in Lipoprotein(a) Levels in the Kazakhstan Population

**DOI:** 10.3390/jcm14176336

**Published:** 2025-09-08

**Authors:** Makhabbat Bekbossynova, Tatyana Ivanova-Razumova, Gulzhan Myrzakhmetova, Saltanat Andossova, Aknur Kali, Aliya Sailybayeva, Timur Saliev

**Affiliations:** 1 Heart Center, Corporate Fund “University Medical Center”, Nazarbayev University, Astana 010000, Kazakhstan; m.bekbosynova@umc.org.kz (M.B.);; 2 Institute of Fundamental and Applied Medical Research, S.D. Asfendiyarov Kazakh National Medical University, Almaty 050000, Kazakhstan

**Keywords:** lipoprotein(a), atherosclerosis, cardiovascular disease, ethnic differences, Kazakhstan, biomarker, ROC analysis, precision-recall curve, risk stratification

## Abstract

**Background:** Lipoprotein(a) (Lp(a)) is a genetically determined lipoprotein that plays an independent role in the development of atherosclerotic cardiovascular disease (ASCVD). Ethnic differences in Lp(a) levels are well-documented, yet regional data from Central Asia, particularly Kazakhstan, remain scarce. **Methods:** We conducted a retrospective, single-center study involving 3727 patients aged ≥ 18 years who underwent Lp(a) testing between January 2023 and June 2024. Participants were stratified by self-reported ethnicity and atherosclerosis status confirmed via coronary angiography. Lp(a) levels were analyzed using immunoturbidimetric assays. **Results:** Elevated Lp(a) levels (≥50 mg/dL) were identified in 19.6% of the total population. While Kazakhs exhibited a slightly higher prevalence of elevated Lp(a) compared to Russians, there were no statistically significant differences in Lp(a) levels across ethnic groups. ROC analysis revealed limited discriminatory power of Lp(a) for diagnosing atherosclerosis (AUC = 0.5464), although PRC analysis showed high sensitivity and precision in both Kazakh and Russian subgroups at lower thresholds. **Conclusions:** Despite modest ethnic variation in Lp(a) distribution, its predictive value for atherosclerosis remains limited as a standalone marker. These findings highlight the need for population-specific thresholds and support incorporating Lp(a) testing in broader cardiovascular risk assessment strategies in Central Asia.

## 1. Introduction

Cardiovascular diseases (CVDs) continues to be the leading cause of death globally, accounting for an estimated 20.5 million deaths (2021), 80% of which occurred in low- and middle-income countries [1]. Atherosclerotic cardiovascular disease (ASCVD), which includes conditions such as coronary artery disease (CAD), peripheral artery disease (PAD), and cerebrovascular disease, represents the most prevalent and fatal form of CVD [2]. While significant progress has been made in managing traditional risk factors such as hypertension, smoking, and hypercholesterolemia, residual cardiovascular risk remains substantial even among patients receiving optimal medical therapy, particularly those with well-controlled low-density lipoprotein cholesterol (LDL-C) [3].

This observation has intensified the search for nontraditional biomarkers that could account for residual cardiovascular risk and offer improved risk stratification. One such biomarker is Lp(a), a genetically determined LDL-like particle covalently linked to apolipoprotein(a) [4]. Lp(a) possesses both atherogenic and thrombogenic properties due to its structural similarity to plasminogen and its ability to carry oxidized phospholipids [5]. Unlike LDL-C, Lp(a) levels are minimally influenced by environmental or lifestyle factors and remain relatively stable over a person’s lifetime. This makes it a desirable candidate for long-term cardiovascular risk assessment [6,7].

Accumulating evidence from prospective cohort studies, genetic studies, and Mendelian randomization trials supports a causal relationship between elevated Lp(a) levels and increased risk of ASCVD [7,8]. Meta-analyses have shown that individuals in the top quintile of Lp(a) levels have up to a 70% higher risk of coronary heart disease compared to those in the lowest quintile [9,10]. Moreover, current clinical guidelines increasingly recommend the inclusion of Lp(a) measurement in routine cardiovascular risk assessment, particularly in individuals with premature ASCVD, a family history of early heart disease, or unexplained elevated LDL-C [11].

A critical challenge in clinical implementation is the marked inter-individual and interethnic variability in Lp(a) concentrations. Studies have shown that individuals of African ancestry have significantly higher Lp(a) levels than those of European or East Asian descent [12,13]. These differences are attributed to genetic diversity, particularly in the LPA gene, which affects the number of kringle IV type 2 repeats and thereby the size and concentration of Lp(a) particles [14]. Consequently, a single universal threshold for Lp(a)-associated risk may not be appropriate across populations.

Despite the growing importance of Lp(a) in cardiovascular medicine, there is a lack of population-specific data from Central Asia. Kazakhstan, a multiethnic country with large populations of ethnic Kazakhs, Russians, and other minorities, presents a unique opportunity to investigate the distribution and clinical relevance of Lp(a) in a previously understudied region [15]. The health systems in this region are also facing a rising burden of cardiovascular morbidity and mortality, underscoring the urgent need for regionally relevant biomarkers to guide prevention efforts.

In this context, the present study aims to (1) characterize Lp(a) levels across different ethnic groups in the Kazakhstani population; (2) evaluate the association between Lp(a) levels and angiographically confirmed atherosclerosis; and (3) assess the diagnostic performance of Lp(a) using ROC and precision-recall curve analyses. By identifying population-specific patterns and thresholds, this study contributes to the evidence base needed to improve personalized cardiovascular risk prediction. It supports the development of tailored screening and prevention strategies for Kazakhstan and similar multiethnic populations.

## 2. Materials and Methods

### 2.1. Study Design and Population

This retrospective, single-center, cross-sectional study was conducted at the National Heart Center (UMC Heart Center, Astana, Kazakhstan) between January 2023 and June 2024. The study included all patients aged 18 years and older who underwent Lp(a) testing during the study period. A total of 3727 individuals were included in the final analysis, with 75.1% (approximately 2799 participants) self-identifying as ethnic Kazakhs.

Patients were excluded if they had incomplete data regarding ethnicity, Lp(a) levels, or coronary angiography results. Individuals receiving lipid-lowering therapy at the time of testing (where such information was available) were also excluded. Although statins are generally not considered to lower Lp(a) concentrations, their use was excluded to avoid potential confounding effects on overall lipid profiles, cardiovascular outcomes, and treatment selection bias. Furthermore, other lipid-lowering agents such as PCSK9 inhibitors and niacin have been reported to influence Lp(a) levels, albeit modestly. Excluding these patients ensured that our analysis reflected untreated baseline Lp(a) values. In cases where multiple Lp(a) measurements were recorded, only the first measurement was included for consistency.

To be eligible for inclusion, participants had to meet the following criteria: age 18 years or older, documented Lp(a) test results, established self-identified ethnicity, and a history of invasive coronary angiography confirming either the presence or absence of atherosclerotic lesions.

To minimize potential variability in Lp(a) measurement, all assays were performed in the same certified laboratory, using standardized reagents and calibration protocols provided by the manufacturer. Measurements were conducted in duplicate, and internal quality controls were applied at each run. Although we excluded patients with incomplete data and those on lipid-lowering therapy, residual confounding from other comorbidities cannot be entirely ruled out. We selected angiographically confirmed atherosclerosis as the outcome variable because this method remains the gold standard for diagnosis and allows more robust correlation between Lp(a) levels and clinically manifest disease.

### 2.2. Biochemical and Clinical Parameters

Lp(a) levels were measured using a standardized immunoturbidimetric assay (Tina-quant Lipoprotein(a), Roche Diagnostics), with results expressed in mg/dL. To account for the skewed distribution of Lp(a) concentrations, log transformation (log Lp(a)) was applied in certain statistical models to reduce bias and improve model performance.

Clinical data were obtained from electronic medical records, and included the following variables: age (calculated as of the date of coronary angiography), sex, self-reported ethnicity, hypertension, diabetes mellitus, coronary artery disease (CAD), peripheral arterial disease (PAD), smoking status, and anthropometric measurements (weight, height, and body mass index [BMI]). Hypertension and diabetes mellitus were defined according to standard diagnostic criteria from the World Health Organization (WHO) and American Heart Association (AHA) guidelines [16]. CAD was defined as the presence of ≥50% luminal stenosis in at least one major coronary artery, confirmed by coronary angiography, consistent with established clinical practice [17,18]. PAD was defined as either a history of lower-limb revascularization, angiographically confirmed atherosclerotic lesions in peripheral arteries, or an ankle–brachial index (ABI) < 0.9, as recommended in international consensus guidelines [19]. Smoking status was classified as current, former, or never smoker, based on patient self-report. BMI was calculated as weight (kg) divided by height squared (m^2^) and categorized according to WHO thresholds.

### 2.3. Ethical Issues

The studies involving humans were approved by the Local Bioethics Committee of the “University Medical Center” corporate fund (Heart Center, Corporate Fund, University Medical Center, Nazarbayev University, Astana, Kazakhstan). The study received approval from the local ethics committee (No. 2023/01-008 dated 14 July 2023). The studies were conducted in accordance with the local legislation and institutional requirements. The participants provided their written informed consent to participate in this study.

### 2.4. Statistics

Data analysis was performed using Stata 14 statistical software, by Stata Corp (College Station, TX, USA). The distribution of continuous variables was assessed for normality using the Shapiro–Wilk (SW) test, which has greater statistical power than the Kolmogorov–Smirnov (KS) test, particularly for moderate sample sizes. Although the overall study population was large, many subgroup analyses (e.g., stratification by ethnicity or angiographic status) included smaller sample sizes, where the SW test provides a more reliable assessment of deviations from normality. To complement the statistical tests, we also performed visual inspection of the distributions using Q–Q plots and histograms to confirm the presence of non-normality. For comparisons between two independent groups, the nonparametric Mann–Whitney U test was applied, while differences among more than two groups were evaluated using the Kruskal–Wallis test. Categorical variables were analyzed using the Pearson χ^2^ test.

To evaluate the prognostic value of Lp(a) in the diagnosis of atherosclerosis, receiver operating characteristic (ROC) curve analysis was conducted, with calculation of the area under the curve (AUC). The optimal cut-off point was determined using Youden’s index (Youden’s J). In addition, precision-recall curve (PRC) analysis was performed, which is more appropriate in the context of class imbalance. The F1 score was used as the optimization metric for PRC.

Differences in Lp(a) threshold values across ethnic subgroups were assessed using bootstrap resampling (n = 1000 iterations) combined with the Mann–Whitney U test. A two-tailed *p*-value of less than 0.05 was considered statistically significant for all analyses.

We also conducted sensitivity analyses to assess the robustness of our findings across different subgroups (e.g., sex, age quartiles). To address potential skewness, Lp(a) was both analyzed as a continuous log-transformed variable and dichotomized at clinically relevant thresholds (≥50 mg/dL vs. <50 mg/dL). Precision-recall curves were applied in addition to ROC curves because of class imbalance between individuals with and without atherosclerosis, thereby providing a more reliable assessment of predictive performance in this clinical setting.

## 3. Results

During the study period at the National Cardiology Center (UMC Heart Center, Astana, Kazakhstan), Lp(a) levels were measured in 3727 patients, representing 0.09% of all patients seen during this time (3727 out of 39,862). Of these, 56.1% were male, and the mean age was 52.3 ± 12.6 years.

Among the patients tested for Lp(a), 1433 underwent coronary angiography, and of these, 1239 (86.4%) were diagnosed with coronary artery disease (CAD). The overall median Lp(a) level was 13.36 mg/dL, with an interquartile range (IQR) of 6.72–35.41 mg/dL. 

By current clinical guidelines, patients were categorized into two groups based on their Lp(a) levels: high Lp(a) (≥50 mg/dL) and low Lp(a) (<50 mg/dL). Elevated Lp(a) levels were observed in 19.6% of the cohort (n = 691), with a median value of 95.19 mg/dL [range: 50.24–451.9 mg/dL].

**Figure 1:**
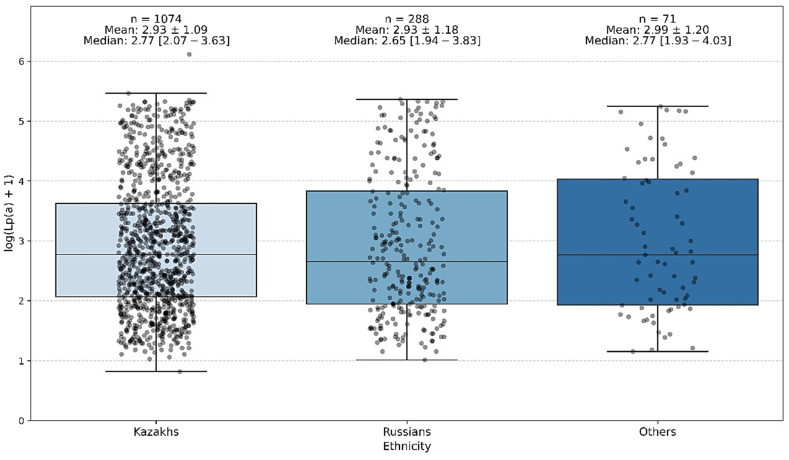
Ethnic variability in lipoprotein(a) concentration, where 0—Kazakhs, 1—Russians, and 2—other nationalities.

Among the patients tested for Lp(a), those with cardiovascular disease (CVD) had significantly higher median Lp(a) levels compared to individuals without CVD (13.1 [3.04–235] vs. 9.19 [2.05–193.49] mg/dL, *p* < 0.001). Figure 1 illustrates the distribution of Lp(a) levels in patients with and without CVD. Notably, individuals with CVD had a lower proportion of cases with Lp(a) < 50 mg/dL, while the proportion of patients with Lp(a) ≥ 50 mg/dL was relatively similar across both groups.

The study also revealed ethnic differences in the prevalence of elevated Lp(a) levels (≥50 mg/dL). Among ethnic Kazakhs, elevated Lp(a) was more common, whereas 18.1% of ethnic Russians showed increased Lp(a) levels. The mean Lp(a) concentration was 34.2 ± 46.17 mg/dL in Kazakhs and 37.7 ± 51.1 mg/dL in Russians (Table 1 and Table 2).

The conducted analysis of interethnic differences showed that the groups differed significantly in age and sex distribution. However, no statistically significant differences were found between the ethnic groups in terms of Lp(a) level.

Thus, the Lp(a) level does not demonstrate any significant ethnic specificity within the studied population of Kazakhstan (Figure 1).

Despite differences in average Lp(a) values, the overall distribution patterns of Lp(a) concentrations did not vary significantly by ethnicity in this cohort (Table 1 and Table 2).

**Figure 2:**
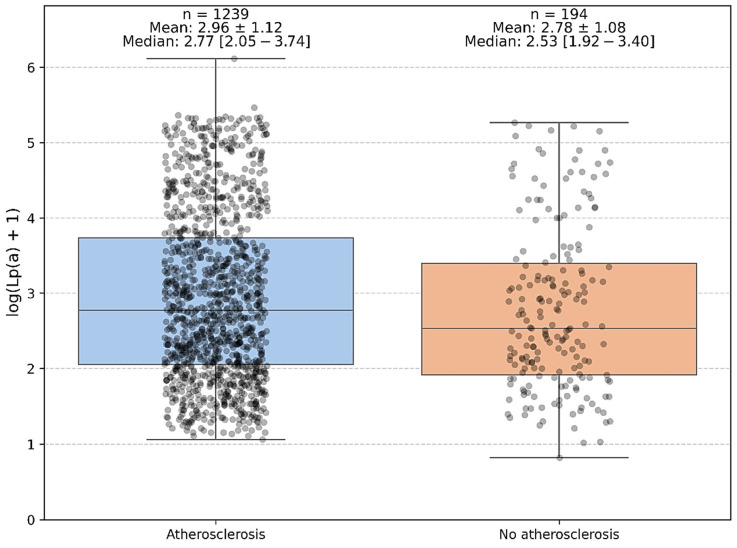
Association of serum lipoprotein(a) with the presence of atherosclerosis.

A comparison of Lp(a) levels between patients with and without established atherosclerosis (defined as angiographically confirmed coronary artery disease [CAD] and/or peripheral arterial disease [PAD]) demonstrated a statistically significant difference. Median Lp(a) concentrations were 14.8 mg/dL (IQR: 7.1–37.4 mg/dL) in patients with atherosclerosis compared with 11.2 mg/dL (IQR: 5.8–28.9 mg/dL) in those without (Mann–Whitney U = 109,027.5, *p* = 0.0374; Figure 2). The corresponding log-transformed values yielded a similar result. While the difference was statistically significant, the calculated effect size was small (r = 0.06), indicating a modest magnitude of association. No additional post hoc subgroup analyses were performed, as the primary comparison was binary (presence vs. absence of atherosclerosis). These findings suggest that elevated Lp(a) levels may be linked with the presence of clinically manifest atherosclerosis, although the effect is modest and likely represents one of multiple contributing risk factors rather than a strong independent predictor.

### 3.1. ROC Analysis: Evaluation of Discriminatory Power of Lp(a) and ROC Analysis by Ethnic Subgroups

**Figure 3:**
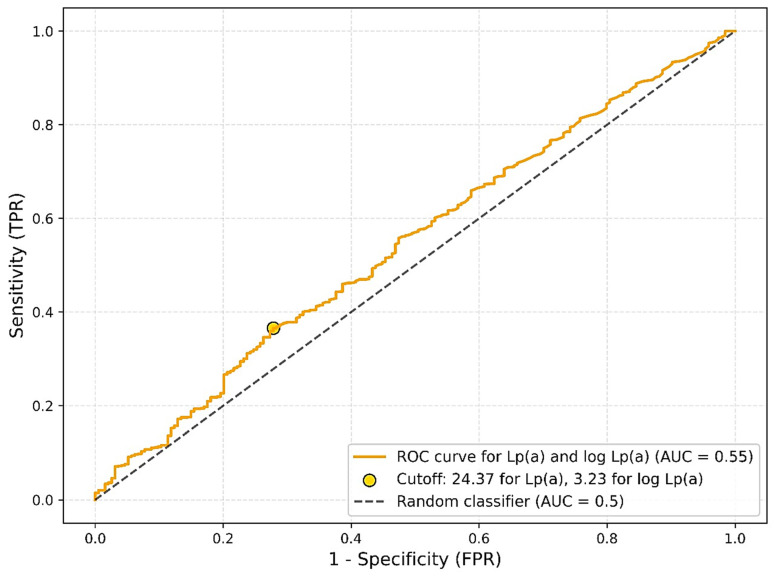
Determination of lipoprotein(a) cutoff using ROC curve.

An evaluation of the diagnostic performance of lipoprotein(a) [Lp(a)] as a single biomarker for predicting the presence of atherosclerosis using receiver operating characteristic (ROC) analysis demonstrated low discriminatory power. The area under the curve (AUC) for Lp(a) was 0.5464, with an optimal cutoff value of 24.37 mg/dL determined by the Youden index. At this threshold, sensitivity was 0.37 and specificity was 0.72. When age and ethnicity were included in the model, the discriminatory ability improved moderately, yielding an AUC of 0.6414 with an optimal cutoff of 0.89, sensitivity of 0.41, and specificity of 0.81. Nevertheless, even with the inclusion of additional covariates, the model did not reach a level of accuracy sufficient for clinical application as a screening tool (Figure 3).

**Figure 4:**
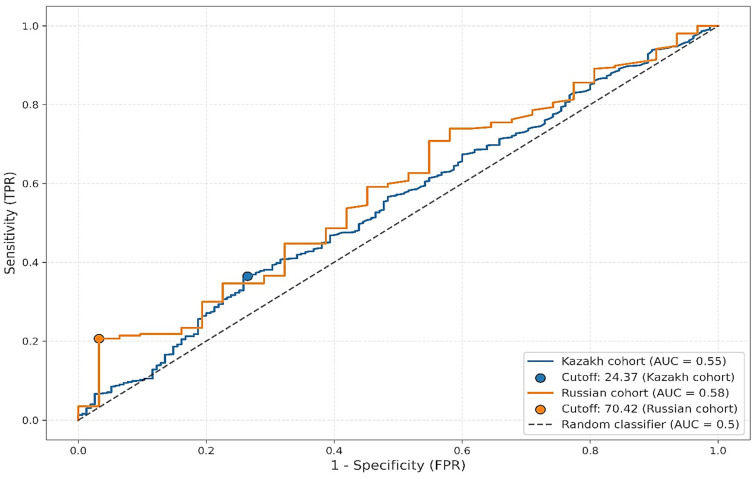
Variability in optimal Lp(a) values between ethnic subgroups.

Further stratified ROC analysis by ethnic groups revealed variability in optimal thresholds and performance metrics (Figure 4). Among Kazakh participants, the AUC was 0.5462 with a cutoff of 24.37 mg/dL, sensitivity of 0.36, and specificity of 0.74. In contrast, among Russian participants, the AUC was slightly higher at 0.5815, with a much higher optimal threshold of 70.42 mg/dL, sensitivity of 0.21, and specificity of 0.97. Despite the observed differences in cutoff values and specificity, the low sensitivity across both groups limits the clinical utility of Lp(a) as a standalone diagnostic marker for atherosclerosis.

### 3.2. Precision-Recall Curve (PRC) Analysis

**Figure 5:**
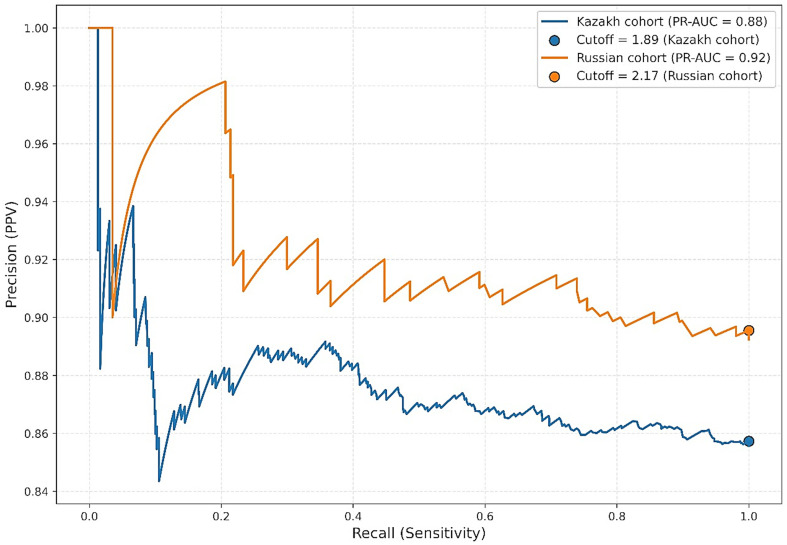
Precision-Recall Curve (PRC) analysis.

In the context of pronounced class imbalance due to the relatively small proportion of patients without angiographically confirmed atherosclerosis, we performed additional evaluation using precision–recall curve (PRC) analysis. PRC is particularly informative when identifying rare positive outcomes. Optimal thresholds were determined by maximizing the F1 score. Among Kazakh participants, the PR-AUC was 0.8755 with an optimal statistical cutoff of 1.89 mg/dL, yielding a precision of 0.86 and recall of 1.00. Among Russian participants, the PR-AUC was 0.9215, with a threshold of 2.17 mg/dL, precision of 0.90, and recall of 1.00. While these thresholds achieved excellent statistical discrimination, they fall well below clinically accepted Lp(a) risk thresholds (≥30–50 mg/dL) and therefore should not be interpreted as clinically actionable cutoffs. Instead, they reflect model optimization in a dataset with class imbalance and should be viewed as methodological findings rather than direct clinical guidance (Figure 5).

### 3.3. Bootstrap Analysis of Thresholds

To further assess the robustness of the identified cutoff values, a bootstrap analysis with 1000 replicates was performed. This analysis revealed a statistically significant difference in the mean optimal cutoff values between the two ethnic subgroups. For Kazakhs, the mean cutoff was 1.95 mg/dL (95% CI: 1.89–2.12), while for Russians, it was 2.22 mg/dL (95% CI: 2.17–2.77), with a *p*-value of less than 0.0001 (Mann–Whitney test). This finding may reflect underlying differences in the pathophysiology of atherosclerosis between the groups or population-level variability in Lp(a) distribution.

### 3.4. Comparison with Clinical Thresholds

It is important to note that the cutoff values identified in this study (≈2 mg/dL) are markedly lower than the thresholds generally applied in clinical practice (e.g., ≥50 mg/dL). This discrepancy does not reflect a unit mismatch or use of a different biochemical fraction, as all Lp(a) measurements in this study were reported in mg/dL, based on immunoassay quantification of total Lp(a) mass. Rather, the divergence arises from the different contexts of application. Clinical thresholds such as ≥50 mg/dL are intended to stratify long-term cardiovascular risk in diverse populations, whereas our model-based thresholds were derived from precision–recall curve optimization within this specific cohort, with pronounced class imbalance. Under such conditions, statistical optimization favors very low cutoffs that maximize sensitivity (recall) but are not clinically actionable. Accordingly, the ≈2 mg/dL threshold should be interpreted as a methodological artifact of the analytic approach, not as a clinical risk threshold.

## 4. Discussion

This study represents one of the first large-scale investigations into the distribution of Lp(a) levels and their association with atherosclerotic cardiovascular disease (ASCVD) in a multiethnic population from Kazakhstan. By evaluating a cohort of 1433 patients who underwent Lp(a) testing, we provide valuable insights into the prevalence of elevated Lp(a), ethnic variability, and its diagnostic performance in detecting angiographically confirmed atherosclerosis.

Our findings demonstrate that approximately 19.6% of patients in the cohort had elevated Lp(a) levels (≥50 mg/dL), consistent with global estimates for Asian populations, which typically range from 10% to 30% [20,21]. This confirms that Lp(a) elevation is a common phenomenon in Kazakhstan and may contribute significantly to cardiovascular risk. Importantly, while slightly higher Lp(a) levels were observed among ethnic Kazakhs compared to Russians, the differences were not statistically significant, suggesting that ethnicity alone does not fully explain individual Lp(a) variability in this population.

Despite the well-established role of Lp(a) in promoting atherogenesis through pro-inflammatory, pro-thrombotic, and lipid-rich pathways, our study found only a modest association between Lp(a) levels and the presence of angiographically confirmed atherosclerosis (*p* = 0.0374). This supports the hypothesis that while Lp(a) may contribute to ASCVD risk, it likely operates in conjunction with other metabolic and genetic factors rather than as a strong independent predictor [22,23]. Furthermore, analysis revealed low diagnostic accuracy, with an AUC of 0.5464, barely above the level of chance. Even after adjusting for age and ethnicity, the model showed only a modest improvement (AUC = 0.6414), falling short of thresholds typically considered clinically useful.

These results reinforce the view that the clinical role of Lp(a) may differ depending on context: while it is a validated marker of long-term cardiovascular risk, its application as a diagnostic biomarker for existing disease remains limited. For Kazakhstan and Central Asia, where premature ASCVD is prevalent, the integration of Lp(a) testing into broader cardiovascular risk models rather than as a standalone test appears most appropriate.

Our findings echo previous research indicating that while Lp(a) is causally linked to long-term ASCVD risk, it may not be reliable as a standalone diagnostic tool for detecting established disease [20]. Instead, Lp(a) may be more appropriately used for lifetime risk stratification, particularly in individuals with a strong family history of premature CVD, unexplained hypercholesterolemia, or statin-resistant residual risk [24].

Interestingly, our analysis showed higher discriminatory value than the ROC analysis. Specifically, both Kazakh and Russian subgroups exhibited excellent recall (sensitivity = 1.00) and high precision (>0.85) at much lower Lp(a) thresholds (~2 mg/dL). These findings suggest that low Lp(a) values may help confidently exclude ASCVD in certain screening contexts. However, such thresholds are markedly lower than those recommended in clinical guidelines, which typically use 30 or 50 mg/dL as risk-stratification cutoffs. This discrepancy highlights the contextual nature of biomarker performance and the need to distinguish between risk prediction and diagnostic utility.

Another important dimension in interpreting our findings is the role of proprotein convertase subtilisin/kexin type 9 (PCSK9) in regulating Lp(a) metabolism. PCSK9 has primarily been studied for its impact on LDL-C via the LDL receptor pathway, yet accumulating evidence indicates that it also influences circulating Lp(a). Clinical trials have consistently shown that PCSK9 inhibitors such as evolocumab and alirocumab lower Lp(a) levels by approximately 20–30% [25,26,27]. This effect is observed independently of LDL-C reduction, suggesting that PCSK9 may play a role in modulating apo(a)-containing lipoprotein clearance or assembly. The biological mechanisms are still under investigation, but potential pathways include enhanced hepatic uptake of Lp(a) particles and indirect modulation of apoB-100 metabolism. The clinical relevance of this PCSK9–Lp(a) interaction has been supported by major outcome trials. In the FOURIER trial, reductions in Lp(a) contributed to incremental decreases in major adverse cardiovascular events, independent of LDL-C lowering [28,29]. Similarly, the ODYSSEY OUTCOMES study demonstrated that patients with elevated baseline Lp(a) derived greater absolute risk reduction from PCSK9 inhibition [29]. These data suggest that therapeutic strategies targeting PCSK9 could be particularly beneficial in populations with a high prevalence of elevated Lp(a), as documented in our cohort.

For Kazakhstan, where approximately one in five individuals exhibited elevated Lp(a) levels, the dual effect of PCSK9 inhibitors on LDL-C and Lp(a) is of particular significance. While Lp(a) was only modestly associated with angiographically confirmed atherosclerosis in our study, the availability of therapies that can simultaneously address two lipid-related risk factors may provide substantial clinical benefit. Future regional studies should therefore explore not only the epidemiology of Lp(a), but also the potential role of PCSK9-targeted therapies in reducing cardiovascular risk across different ethnic groups. Incorporating PCSK9–Lp(a) interactions into clinical decision-making frameworks could help refine patient stratification and guide the adaptation of international lipid management guidelines to local practice.

Our bootstrap analysis confirmed statistically significant differences in optimal Lp(a) thresholds between ethnic groups, further supporting the notion that population-specific calibration is essential for meaningful interpretation. Nonetheless, the clinical relevance of these differences remains unclear, especially given the overlapping distributions and absence of corresponding variation in disease prevalence. It is also possible that the observed ethnic variability reflects broader socio-demographic or environmental influences, such as differences in access to healthcare, diet, physical activity, or comorbidities, rather than purely genetic factors.

Furthermore, the observed high burden of CAD and other ASCVDs in this relatively young patient cohort is consistent with regional reports suggesting earlier onset and more aggressive disease progression in Central Asian populations. This reinforces the urgency of identifying high-risk individuals earlier and integrating biomarkers such as Lp(a) into comprehensive cardiovascular prevention strategies.

Another point worth noting is the limited public and clinical awareness of Lp(a) in Kazakhstan and the broader Central Asian region. Routine Lp(a) testing remains uncommon, and healthcare providers may not be fully informed about its clinical relevance or appropriate interpretation. Our findings underscore the need for education, guideline development, and increased accessibility to standardized Lp(a) assays, especially as targeted therapies, such as antisense oligonucleotides and RNA interference drugs, are currently in clinical development and could soon offer new treatment options for patients with elevated Lp(a).

From a public health perspective, our findings highlight the need to develop population-specific cardiovascular risk assessment frameworks that incorporate Lp(a) while considering regional demographics, genetic diversity, and healthcare infrastructure. Importantly, the modest ethnic variability observed here suggests that broad, ethnicity-specific thresholds may not be as impactful in Kazakhstan as they are in other regions. Future longitudinal studies will be essential to determine whether Lp(a)-targeted interventions, particularly with novel therapies such as antisense oligonucleotides, can meaningfully alter cardiovascular outcomes in Central Asian populations.

In summary, our study supports the inclusion of Lp(a) as part of a comprehensive cardiovascular risk assessment framework in Kazakhstan, particularly in individuals with unexplained or premature ASCVD. However, the limitations of Lp(a) as a diagnostic tool for established atherosclerosis must be acknowledged, and further prospective studies are needed to clarify its role and optimal use in clinical practice. Importantly, any application of Lp(a) testing should consider local population characteristics, including genetic background, disease burden, and health system infrastructure.

## 5. Study Limitations

This study has several limitations. As a retrospective, single-center analysis, the results may be subject to selection bias and may not fully represent the general population of Kazakhstan. Ethnicity was self-reported and not genetically verified, which may introduce misclassification bias. Additionally, we lacked data on other potential confounders such as lipid-lowering therapies, inflammatory markers, and genetic polymorphisms that influence Lp(a) levels. The study did not account for patients with secondary causes of elevated Lp(a), and the cross-sectional design precludes causal inference regarding Lp(a) and disease development.

## 6. Conclusions

This study provides new evidence on the distribution and clinical relevance of Lp(a) in Kazakhstan’s multiethnic population. Elevated levels (≥50 mg/dL) were found in about one in five individuals, consistent with the global burden of genetically determined dyslipidemia. Although modest differences between ethnic groups were observed, these were not statistically significant. Lp(a) showed only a weak association with angiographically confirmed atherosclerosis and limited diagnostic performance, with ROC analysis demonstrating poor discrimination even after covariate adjustment. Precision–recall analysis suggested that very low Lp(a) levels may aid in ruling out disease in low-prevalence settings, though clinical applicability remains limited. Overall, Lp(a) should not be considered a standalone diagnostic marker for atherosclerosis but remains a relevant risk factor for long-term cardiovascular disease. These findings support broader implementation of Lp(a) testing in Kazakhstan, encourage further research into its ethnic and clinical variability, and highlight the need for regional guidelines tailored to local practice.

## Figures and Tables

**Table 1:** Distribution of cardiovascular conditions in the patient cohort by sex and ethnicity (Total n—1433).

Variable	Statistic	Overall	Kazakhs	Russians	Other Ethnicities	Chi^2^ Statistic	Chi^2^ *p*-Value (FDR-BH)	Cramér’s V
sex	female	530 (37.0%)	385 (35.8%)	126 (43.8%)	19 (26.8%)	9.44	0.017862	0.081
	male	903 (63.0%)	689 (64.2%)	162 (56.2%)	52 (73.2%)			
	Group 0 vs. 1	OR = 1.39 [1.07–1.81], *p* = 0.0327						
	Group 0 vs. 2	OR = 0.65 [0.38–1.12], *p* = 0.1260						
	Group 1 vs. 2	OR = 0.47 [0.26–0.83], *p* = 0.0304						
atherosclerosis	Class 0 (no)	194 (13.5%)	155 (14.4%)	31 (10.8%)	8 (11.3%)	2.94	0.229981	0.045
	Class 1 (yes)	1239 (86.5%)	919 (85.6%)	257 (89.2%)	63 (88.7%)			

**Table 2:** Age and lipoprotein(a) levels in the patient cohort stratified by ethnicity (Total n—1433).

Variable	Statistic	Overall	Kazakhs	Russians	Other Ethnicities	Kruskal- Wallis H	KW *p*-Value	η^2^
Age [years]	Mean ± SD	61.29 ± 9.76	60.77 ± 9.63	62.45 ± 10.06	64.37 ± 9.79	19.75	0.000103	0.012
	Median [Q1–Q3]	62.00 [56.00–68.00]	61.00 [55.00–67.00]	64.00 [57.00–69.00]	65.00 [60.00–71.00]			
	Min–Max	19.00–89.00	19.00–89.00	34.00–86.00	38.00–87.00			
	Dunn: Group 0 vs. 1	*p* = 0.0020, r = 0.092						
	Dunn: Group 0 vs. 2	*p* = 0.0025, r = 0.095						
	Dunn: Group 1 vs. 2	*p* = 0.1820, r = 0.070						
Lp(a) [mg/dL]	Mean ± SD	35.18 ± 47.39	34.22 ± 46.17	37.77 ± 51.15	39.29 ± 49.77	0.34	0.843022	−0.001
	Median [Q1–Q3]	14.74 [6.66–38.82]	14.97 [6.89–36.53]	13.22 [5.98–45.07]	14.88 [5.88–55.33]			
	Min–Max	1.27–451.90	1.27–451.90	1.76–212.35	2.17–188.95			

## Data Availability

The data presented in this study are available on request from the corresponding author.
